# Bacteraemia, Malaria, and Case Fatality Among Children Hospitalized With Fever in Dar es Salaam, Tanzania

**DOI:** 10.3389/fmicb.2020.02118

**Published:** 2020-09-10

**Authors:** Sabrina J. Moyo, Joel Manyahi, Bjørn Blomberg, Marit Gjerde Tellevik, Nahya Salim Masoud, Said Aboud, Karim Manji, Adam P. Roberts, Kurt Hanevik, Kristine Mørch, Nina Langeland

**Affiliations:** ^1^Department of Clinical Science, University of Bergen, Bergen, Norway; ^2^Department of Microbiology and Immunology, Muhimbili University of Health and Allied Sciences, Dar es Salaam, Tanzania; ^3^Norwegian National Advisory Unit on Tropical Infectious Diseases, Haukeland University Hospital, Bergen, Norway; ^4^Department of Microbiology, Haukeland University Hospital, Bergen, Norway; ^5^Department of Paediatrics, Muhimbili University of Health and Allied Sciences, Dar es Salaam, Tanzania; ^6^Department of Tropical Disease Biology, Liverpool School of Tropical Medicine, Liverpool, United Kingdom

**Keywords:** bacteraemia, malaria, antimicrobial resistance, *P. falciparum*, Tanzania

## Abstract

**Background:**

Febrile illness is the commonest cause of hospitalization in children <5 years in sub-Saharan Africa, and bacterial bloodstream infections and malaria are major causes of death.

**Methods:**

From March 2017 to July 2018, we enrolled 2,226 children aged 0–5 years hospitalized due to fever in four major public hospitals of Dar es Salaam, namely, Amana, Temeke, and Mwananyamala Regional Hospitals and Muhimbili National Hospital. We recorded social demographic and clinical data, and we performed blood-culture and HIV-antibody testing. We used qPCR to quantify *Plasmodium falciparum* parasitaemia and Matrix-Assisted Laser Desorption/Ionization-Time of Flight (MALDI-TOF) to identify bacterial isolates. Disk diffusion method was used for antimicrobial susceptibility testing.

**Results:**

Nineteen percent of the children (426/2,226) had pathogens detected from blood. Eleven percent (236/2,226) of the children had bacteraemia/fungaemia and 10% (204/2,063) had *P. falciparum* malaria. Ten children had concomitant malaria and bacteraemia. Gram-negative bacteria (64%) were more frequent than Gram-positive (32%) and fungi (4%). Over 50% of Gram-negative bacteria were extended-spectrum beta-lactamase (ESBL) producers and multidrug resistant. Methicillin resistant *Staphylococcus aureus* (MRSA) was found in 11/42 (26.2%). The most severe form of clinical malaria was associated with high parasitaemia (>four million genomes/μL) of *P. falciparum* in plasma. Overall, in-hospital death was 4% (89/2,146), and it was higher in children with bacteraemia (8%, 18/227) than malaria (2%, 4/194, *p* = 0.007). Risk factors for death were bacteraemia (*p* = 0.03), unconsciousness at admission (*p* < 0.001), and admission at a tertiary hospital (*p* = 0.003).

**Conclusion:**

Compared to previous studies in this region, our study showed a reduction in malaria prevalence, a decrease in in-hospital mortality, and an increase in antimicrobial resistance (AMR) including ESBLs and multidrug resistance. An increase of AMR highlights the importance of continued strengthening of diagnostic capability and antimicrobial stewardship programs. We also found malaria and bacteraemia contributed equally in causing febrile illness, but bacteraemia caused higher in-hospital death. The most severe form of clinical malaria was associated with *P. falciparum* parasitaemia.

## Introduction

Bloodstream infections (BSI) are the commonest causes of hospital admissions and deaths in children in sub-Saharan Africa (Bahwere et al., 2001; Campbell et al., 2004; Peters et al., 2004; Bryce et al., 2005; Feikin et al., 2011; Maze et al., 2018). Severe BSI due to bacteria and malaria parasites are often difficult to differentiate clinically (Evans et al., 2004) especially where semi-immune children may have malaria parasites in their blood without clinical disease. Therefore, to guide management of these patients, a more specific test that can identify level of parasitemia and support the clinical differentiation between severe malaria, malaria causing disease, and subclinical malaria controlled by immunity is important. A recent study (Imwong et al., 2015) has shown that plasma qPCR can play a role in detecting and differentiating severe malaria from non-severe malaria by quantifying *Plasmodium falciparum* in plasma samples.

Recent efforts in scaling up malaria control programs such as increased use and availability of rapid diagnostic tests (RDT), distribution of free insecticide-treated bed nets, and change to more effective malaria treatment guidelines in sub-Saharan Africa have resulted in a substantial decline of malaria-associated morbidity and mortality (O’Meara et al., 2010; Smithson et al., 2015; Hershey et al., 2017; Kayentao et al., 2018).

Determining bacterial or fungal infections in BSI is fundamental to management of severe BSI based on etiology. This is challenging in low-income countries because easily available and affordable diagnostic services for BSI other than malaria is limited by both infrastructure and cost (Archibald and Reller, 2001; Petti et al., 2006). As a result, clinicians in low-income countries lack information on local causes of severe BSI and rely on empirical treatment guided by WHO Integrated Management of Childhood Illnesses (IMCI) guidelines. Although IMCI is useful, it may underestimate or overestimate the likelihood of certain causes of disease, risking poor clinical outcomes and the promotion of antimicrobial resistance. Appropriate management of severe BSI in children is also required to minimize the spread of antimicrobial resistance (AMR).

Microbial etiology of febrile illness varies from bacteria, viruses, parasites, or fungi, between children attending outpatient health care facilities versus in hospitals (Maze et al., 2018), and geographically (Maze et al., 2018; Moreira et al., 2018; Shrestha et al., 2018).

In the Dar es Salaam region of Tanzania, a previous study reported causes of febrile illness among children attending out-patient clinics only (D’Acremont et al., 2014). Three other studies were conducted in a single tertiary referral hospital, Muhimbili National Hospital, two of which focused solely on neonatal sepsis (Blomberg et al., 2005; Mhada et al., 2012; Mkony et al., 2014). None of these studies used plasma qPCR to detect and quantify *P. falciparum* malaria.

With the reduction of malaria in mind, information is required, first on the current causes of severe febrile illness and death among hospitalized children, second on the role of *P. falciparum* qPCR to quantify parasitaemia and differentiate severe from non-severe clinical malaria, and lastly, the current status of antimicrobial resistance. The present study provides updated information on the contribution of bacteria, fungi, and malaria in severe febrile illness among children admitted in four major hospitals of Dar es Salaam, Tanzania. We also report the risk factors for in-hospital death. In addition, the study shows the association of clinical malaria severity and the levels of *P. falciparum* parasitemia, using plasma qPCR. We hypothesized that there is widespread antimicrobial resistance in pathogens causing BSI. Our findings showed that there is an increase in the rate of antimicrobial resistance, extended spectrum beta-lactamases, and multidrug resistance. The information will increase clinical awareness of the emerging public health problem of AMR.

## Materials and Methods

### Study Sites and Population

This hospital-based cross-sectional study was conducted from March 2017 to July 2018 in Dar es Salaam, Tanzania. The study consecutively included children aged ≤5 years who were hospitalized because of fever (≥37.5°C) at three regional hospitals, Amana, Temeke, and Mwananyamala, and a tertiary hospital, Muhimbili National Hospital (MNH). These are the main public hospitals that serve approximately five million people living in the Dar es Salaam region. The entry point of the study was admission of the patient, and the end point was at discharge of the patient from the hospital, referral to other facilities, and death.

### Collection of Demographic and Clinical Information

Parents/caretakers of study participants, who consented to participate in the study, were interviewed, and demographic information was gathered using the electronic case report form (eCRF) REDCap. Information recorded were date of birth, sex of the child, duration of fever, history and duration of convulsions, vomiting, cough, diarrhea and its duration, antibiotic use during 1 month before the current admission, and history of hospital admissions during the last 6 months. The attending physician performed clinical examination and recorded the following information: level of consciousness, pulse rate, respiratory rate, neck stiffness, irritability, and jaundice. Hemoglobin level was recorded when available. Information was recorded on any antibiotic or antimalarial prescribed by the attending physician, and whether the child had received antibiotics or antimalarials before blood culture was taken. Outcome was recorded as discharged, transferred to another hospital, or died.

### Specimen Collection and Processing

A total of 1–5 mL of blood was collected from each child within 24 h of admission. 1–2 mL of blood was inoculated in a pediatric blood culture bottle and incubated at 35°C using the BACTEC 9050^®^ (Becton–Dickinson, Sparks, MD, United States) culture system for a maximum of 5 days unless flagged positive earlier. The remaining blood was aliquoted, one portion of whole blood was added to Tempus blood RNA tubes (Thermo Fisher Scientific, Waltham, MA, United States), and the rest if any was centrifuged at 3,000 rpm for 10 min at 4°C to obtain plasma and stored at −80°C until further analysis. Blood remaining after aliquoting was used for HIV rapid testing as per national rapid HIV testing algorithm, SD bioline HIV-1/2 3.0 (Standard Diagnostics Inc., South Korea) followed by Uni-Gold^TM^ HIV (Trinity Biotech Manufacturing Ltd., Bray, Ireland).

### Detection and Identification of Bacteria and Fungi

Positive blood cultures were sub-cultured on sheep-blood, MacConkey, chocolate, and Sabouraud Dextrose agar (SDA) plates. Standard microbiological methods such as conventional biochemical tests and analytical profile index (API) (bioMérieux SA, Marcy l’Etoile, France) were used for identifying bacterial isolates. Fungal isolates that grew on SDA were Gram stained, and for yeast cells germ-tube test was performed. Final identification of both bacterial and fungal isolates was done with MALDI-TOF MS using the Microflex LT instrument and MALDI Biotyper 3.1 software (Bruker Daltonics, Bremen, Germany).

### Antimicrobial Susceptibility Testing

Antimicrobial susceptibility testing was done by disk diffusion technique according to guidelines and breakpoints of the Clinical Laboratory Standards Institute (CLSI; CLSI, 2019). Isolates showing intermediate susceptibility were categorized as resistant. Screening of ESBL phenotype was performed using cefotaxime and ceftazidime disk, and those with reduced susceptibility were confirmed with the BBL Sensi-Disc ESBL Confirmatory Test Disks (Becton Dickinson, Sparks, MD, United States). Screening for MRSA was done using cefoxitin disk, and resistant *Staphylococcus aureus* isolates were confirmed by the presence of *mecA* using PCR as previously described (Moyo et al., 2014). Screening and confirmation of vancomycin resistance for *S. aureus* and *Enterococcus* spp. was done using *E*-test (bioMérieux). Quality control strains were included for all screening tests and PCR.

### Extraction of Total Nucleic Acid (TNA)

Pathogen Universal Kits (Roche Diagnostics) for total nucleic acid (TNA) were used for extraction of both DNA and RNA from plasma and whole blood, on MagNA Pure 96 instrument (Roche Diagnostics GmbH, Mannheim, Germany). Input volume of plasma and/whole blood was 500 μL, extracted TNA (100 μL) was stored at −80°C.

### Detection of Malaria and Parasite Quantification

*Plasmodium falciparum* was detected and quantified using qPCR. The target used for detection of *P. falciparum* was *var*ATS, using primers, probes, and conditions described previously (Hofmann et al., 2015). Master mix with primers and probes dispensed on a 384 plate was provided by Eurogentec (Eurogentec, Liège, Belgium). Briefly a total reaction volume of 13.3 μL was used, of which master mix was 9.3 μL of Takyon^TM^ Master Mix (Eurogentec, Liège, Belgium) and template volume was 4 μL. The final concentration of primer and probe were 800 and 400 nM, respectively. PCR was done using the Light Cycler 480 II Instrument (Roche Diagnostics, Mannheim, Germany). Each run included a positive control and multiple no-template controls. A plasmid with synthetic *var*ATS insert was included (Blue Heron Biotech, Bothell, WA, United States) for PCR and for quantification. DNA quantity for samples with *P. falciparum* DNA less than the Limit of Quantification (LOQ) was set to be equal to or less than the LOQ (estimated to ≤0.05 genomes/μL). The efficiency (using the formula *E* = 10^–1/slope^ − 1) was 91.2%, and error value (E) of the assay was 0.012.

### Statistical Analysis

Data collected and entered in REDCap was exported to Statistical Package for Social Sciences (SPSS) version 24.0, which was used for data analysis. Categorical data were displayed using frequencies and percentages, and continuous data were displayed using the mean/median and interquartile range (IQR). Chi square and Fischer’s exact test were used to determine association between categorical variables. The risk factors for in-hospital mortality, associations between bacteraemia/malaria, and the demographic/clinical variables were analyzed using univariable and multivariable logistic regression analyses controlling for age and gender of the children. Association between *P. falciparum* malaria parasitaemia by qPCR and clinical malaria severity scores was assessed by ANOVA. For all analyses, a *P*-value of <0.05 was considered statistically significant.

## Results

### The Study Population

**FIGURE 1:**
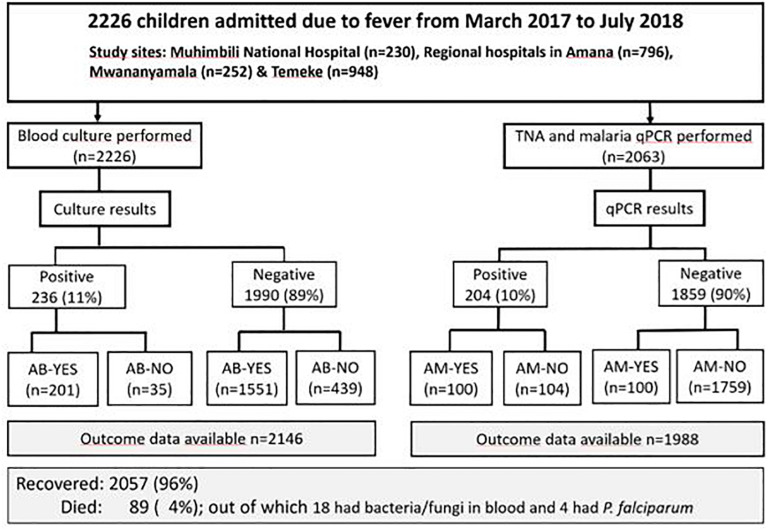
The distribution of the study population. *Sample size for malaria (*N* = 1988); AB, antibiotic treatment received on admission, AM, antimalaria treatment received on admission.

The study included 2,226 children under 5 years of age. Of these 1,299 (58%) were males. The majority of children were below 2 years of age (1,821/2,226, 82%). The mean and median age were 11.09 and 2.04 months, respectively. Figure 1 shows the distribution of study participants from the different study sites. Temeke Regional Referral Hospital accounted for many participants, 948/2,226 (43%), followed by Amana Regional Referral Hospital, 796/2,226 (36%).

HIV test results were available for 1,169/2,226 (53%), and 4% (40/1,169) tested positive for HIV. Symptoms among patients with malaria and bacteraemia are shown in Supplementary Table 1.

Of the 2,226 children admitted with fever, 33.6% (747/2,226) had symptoms representing specific syndromes as follows: gastroenteritis (diarrhea 10.9% [243/2,226]); meningitis/neck stiffness, 2.7% (59/2,226); and respiratory tract infection/cough, 20.0% (445/2,226).

### Prevalence of Bacteria, Fungi, and *P. falciparum* in Blood

**FIGURE 2:**
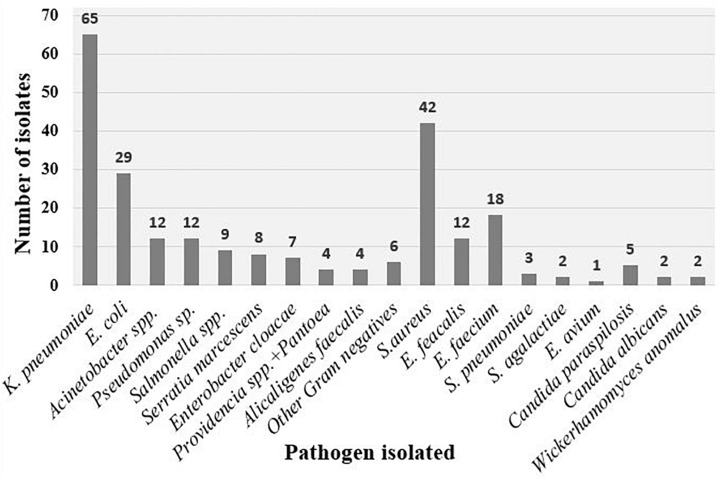
Pathogens isolated from blood culture. Other Gram-negative bacteria include two *Proteus mirabilis* and one of each of the following; *Brevundimonas diminuta, Ralstonia pickettii, Microbacterium aurum*, and *Ochrobactrum tritici.*

At least one pathogen was detected in blood from 19% (426/2,226) of the patients. A total of 243 bacteria and fungi were isolated from 236/2,226 children (11%) (Figure 1). The majority of pathogens isolated were Gram-negative bacteria (GNB), (*n* = 156, 64%), followed by Gram-positive bacteria (GPB) (*n* = 78, 32%) and fungi (*n* = 9, 4%). Figure 2 shows distribution of bacteria and fungi isolated.

Gram-negative bacteria isolated were *Klebsiella pneumoniae* (42%), *Escherichia coli* (19%), *Pseudomonas* spp. (8%), *Acinetobacter* spp. (8%), *Salmonella* spp. (6%), *Serratia marcescens* (5%), *Enterobacter cloacae* (5%), *Alcaligenes faecalis* (3%), *Pantoea* and *Providencia* spp. (3%), other Gram-negative bacteria (6/156, 3.85%) were *Proteus mirabilis* (*n* = 2), *Brevundimonas diminuta* (*n* = 1), *Ralstonia pickettii* (*n* = 1), *Microbacterium aurum* (*n* = 1), and *Ochrobactrum tritici* (*n* = 1).

Gram-negative bacteria isolated were *S. aureus* (54%), *E. faecalis* (15%), *Enterococcus faecium* (23%), *S. pneumoniae* (4%), and *Streptococcus agalactiae* (3%) and *Enterococcus avium* (1%).

Fungi isolated were *Candida parapsilosis* (56%), *Candida albicans* (22%), and *Wickerhamomyces anomalus* (22%). In this study we performed single blood culture, therefore we could not confirm coagulase negative *Staphylococcus* as a cause of the blood stream infection. Coagulase-negative *Staphylococcus*, *Bacillus*, and *Micrococcus* spp. were regarded as likely contaminants and excluded from analysis. These contaminants were found in 4.1% (92/2,226).

Plasma and whole blood samples were available for 1,811 and 252 children, respectively, and total nucleic acid (TNA) was extracted from these 2,063 children. The prevalence of *P. falciparum* malaria was 10% (204/2,063).

### Malaria Severity by Clinical Criteria and qPCR of *P. falciparum* From Plasma

To categorize severe and non-severe malaria we used the presence of one or more of the following severity criteria following World Health Organization (WHO) guidelines (World Health Organization [WHO], 2014): jaundice, high respiratory rate as defined by WHO, convulsions, unconsciousness with Blantyre Coma Scale < 3, and anemia with hemoglobin < 5 g/dL. Deaths and parasite quantity among children categorized with increasing number of severity scores, divided in four groups, are shown in Table 1. The median quantity of *P. falciparum* in plasma measured by qPCR (expressed as genomes/μL) was significantly higher in children with 3 or more severity criteria compared to the other groups.

**TABLE 1:** The quantity (median value) of *Plasmodium falciparum* in plasma in genomes/μL for the four groups and children who died in each group, and *P*-value for each group compared with group 3.

**Malaria severity score**	***N***	**Number of deaths**	**Quantity of *P. falciparum* genomes/μL (range)**	***P*-value**
**Group 0**	94	0	7.56 × 10^5^ (0.46 × 10^0^–1.77 × 10^7^)	0.01
Non-severe malaria				
**Group 1**	61	0	5.77 × 10^5^ (0.27 × 10^0^–7.78 × 10^6^)	0.008
Severe malaria with 1 criterium				
**Group 2**	34	1	9.08 × 10^5^ (1.40 × 10^0^–2.53 × 10^7^)	0.041
Severe malaria with 2 criteria				
**Group 3**	15	3	4.07 × 10^6^ (1.02 × 10^1^–37.2 × 10^7^)	**–**
Severe malaria with 3 or more criteria				

### Bacteria and Malaria Co-infection

Among children with malaria, 5% (10/204) had co-infection with bacteraemia. Bacteria isolated among these children were *Serratia marcescens* (*n* = 2), *K. pneumoniae* (*n* = 2), and one of each of the following bacteria: *E. coli*, *Acinetobacter ursingii*, *Alcaligenes faecalis*, *Ochrobactrum tritici*, *E. faecium*, and *S. aureus.* Of the 10 children with bacteria-malaria co-infection, 40% (4/10) had clinically severe disease and high malaria parasitaemia by qPCR, and one of these four children died.

### Treatment Received on Admission

Of 2,226 children included, 10% (216/2,226) received anti-malarial treatment and 79% (1752/2,226) antibiotic treatment on admission, before blood culture was taken (Figure 1). Of 200 children who were treated with anti-malarials, 50% (100/200) had malaria positive qPCR results. Among malaria qPCR positive children, as many as 51% (104/204) did not receive antimalarials at admission. Of those who received antibiotics, only 12% (201/1,752) had a positive blood culture. Among children with bacteraemia, 15% (35/236) did not receive antibiotic on admission (Figure 1).

### Association Between Malaria/Bacteraemia and Demographic/Clinical Characteristics

Table 2 summarizes associations between malaria or bacteraemia/fungaemia and social demographic or clinical characteristics in multivariable logistic regression model analyses. The prevalence of bacteria and fungi was highest (14%) in children below the age of 3 months (*P*-value < 0.001, OR 2.81, 95%CI 1.73–4.58). The median age for malaria infection was 27 months. Malaria prevalence was significantly increasing with age, with the highest prevalence (31%) among children older than 25 months (*P*-value < 0.001, OR 20.04, 95% CI 12.72–31.58). Unconsciousness was also associated with malaria with P value 0.03, OR 1.83, and 95% CI (1.07–3.13). In univariable analysis, severe anemia, convulsions, reduced consciousness, and vomiting were associated with malaria, as shown in Supplementary Table 1.

**TABLE 2:** Multivariable analyses of bacteraemia/fungaemia and malaria and social demographic/clinical characteristics of children admitted with fever from March 2017 to July 2018 in Dar es Salaam, Tanzania.

**Demographic/clinical characteristics**	**Malaria (*n* = 2063)**			**Bacteraemia/fungaemia (*n* = 2,226)**		
	***N***	***n* (%)**	***P*-value; OR (95% CI)**	***N***	***n* (%)**	***P*-value; OR (95% CI)**
**Age in months**						
0–3	1115	31 (2.8)	1	1214	172 (14.2)	**<0.001; 2.75 (1.72**–**4.40)**
4–6	111	10 (9.0)	**0.001;** 3.49 (1.65–7.35)	122	10 (8.2)	0.35; 1.45 (0.66–3.16)
7–11	212	15 (7.1)	**0.004;** 2.58 (1.36–4.89)	220	12 (5.5)	0.84; 0.93 (0.45–1.92)
12–24	285	39 (13.7)	**<0.001;** 5.43 (3.30–8.92)	304	20 (6.6)	0.69; 1.14 (0.61–2.13)
≥25	376	116 (30.9)	**0.001;** 15.27 (9.92–23.49)	366	22 (6.0)	1
**Sex**						
Male	1205	127 (10.5)	0.30; 0.84 (0.61–1.16)	1299	143 (11.0)	0.34; 0.87 (0.66–1.15)
Female	858	77 (9.0)	1	927	93 (10.0)	1
**Consciousness**						
Unconscious	119	32 (26.9)	**0.004; 2.19 (1.29**–**3.71)**	136	18 (13.2)	0.09; 1.69 (0.92–3.10)
Awake	1944	172 (8.8)	1	2090	218 (10.4)	1
**Convulsions**						
Yes	307	54 (17.6)	0.63; 1.11 (0.73–1.66)	339	35 (10.3)	0.95; 1.02 (0.65–1.59)
No	1756	150 (8.5)	1	1887	201 (10.7)	1
**Jaundice**						
Yes	59	10 (16.9)	0.12; 1.87 (0.85–4.14)	63	8 (12.7)	0.98; 0.99 (0.45–2.18)
No	2004	194 (9.7)	1	2163	228 (10.5)	1

*OR, odds ratio; CI, confidence interval; Confidence intervals that do not overlap the null value of OR = 1 are shown in bold.*

### In-Hospital Death and Risk Factors

Outcome data were available for 2,146 and 1,988 children tested for bacteraemia/fungaemia and malaria qPCR, respectively. Of 2,146 children, 89 (4%) died while 2,057 (96%) improved and were discharged from the hospital. Of children who died, 25% (22/89) had pathogens detected in blood; 18 had bacteria/fungi and four had *P. falciparum*. Outcome was not known for 80 children (4%); 58 were referred to a different hospital because their condition worsened, while for 22 children information was not recorded. Table 3 summarizes risk factors for in-hospital mortality in the multivariable regression analyses. Mortality varied at different study sites, with MNH showing higher mortality compared to the rest of the study sites. Independent risks of death in multivariable analysis were admission at a tertiary hospital (MNH), bacteraemia, and unconsciousness on admission. In the univariable analysis, in-hospital death was significantly higher among HIV-infected children 6/37 (16.2%) vs. non-HIV-infected children 71/1,919 (3.7%), *P*-value 0.003; OR 3.68, 95% CI (1.47–9.21).

**TABLE 3:** Risk factors for in-hospital death among children admitted with fever from March 2017 to July 2018 in Dar es Salaam, Tanzania.

**Demographic/clinical characteristics**	**Death**	**Multivariable analysis**
		***n* = 1,988**
	***n* (%)**	***P*-value; OR (95% CI)**
**Age of the child in months**		
0–3 (*n* = 1185)	43 (3.6)	0.13; 1.98 (0.82–4.80)
4–6 (*n* = 113)	6 (5.3)	0.16; 2.40 (0.70–8.22)
7–11 (*n* = 212)	13 (6.1)	0.29; 1.69 (0.63–4.56)
12–24 (*n* = 284)	15 (5.3)	0.21; 1.83 (0.71–4.70)
≥25 (*n* = 352)	12 (3.4)	1
**Sex**		
Female (*n* = 898)	40 (4.5)	0.59; 1.15 (0.69–1.89)
Male (*n* = 1,248)	49 (3.9)	1
**Study site**		
Mwananyamala (*n* = 236)	1 (0.4)	1
MNH (*n* = 220)	25 (11.4)	**0.003; 20.99 (2.72**–**161.85)**
Amana (*n* = 751)	21 (2.8)	0.28; 3.19 (0.39–26.22)
Temeke (*n* = 939)	42 (4.5)	0.09; 5.97 (0.78–46.02)
**Malaria**		
Positive (*n* = 194)	4 (2.1)	**0.02; 0.25 (0.08**–**0.79)**
Negative (*n* = 1,794)	74 (4.1)	1
**Bacteraemia/fungaemia**		
Positive (*n* = 227)	18 (7.9)	**0.03; 2.04 (1.09**–**3.83)**
Negative (*n* = 1,919)	71 (3.7)	1
**Convulsions**		
Yes (*n* = 315)	33 (10.5)	0.74; 0.89 (0.44–1.79)
No (*n* = 1,831)	56 (3.1)	1
**Consciousness**		
Unconscious (*n* = 122)	39 (32.0)	**<0.001; 25.54 (12.07**–**54.01)**
Awake (*n* = 1,543)	37 (2.4)	**1**
**Jaundice**		
Yes (*n* = 60)	7 (11.7)	0.49; 1.47 (0.49–4.43)
No (*n* = 2086)	82 (3.9)	1

*OR, odds ratio; CI, confidence interval; confidence intervals that do not overlap the null value of OR = 1 are shown in bold.*

Four children with malaria died. All had severe malaria according to clinical criteria, and in addition high parasitaemia of >1.6 million *P. falciparum* genomes/μL (Table 1). All were unconscious on admission. One was co-infected with *K. pneumoniae*. All four were HIV negative. Three of the children were treated with artesunate, while the child with bacterial co-infection did not receive anti-malarial treatment.

Among the 18 children who died and had bacteraemia, 7/18 (39%), 4/18 (22%), and 2/18 (11%) were infected with *K. pneumoniae*, *E. coli*, and *S. aureus*, respectively. Additionally, two children were infected with *Pseudomonas* spp., one with *Acinetobacter baumannii*, one with *S. marcescens*, and one with *Streptococcus pneumoniae.*

Of the 18 children who died, 16 (88%) received antibiotic treatment. Antibiotics prescribed to these children were a combination of either amoxycillin/clavulanic acid or ceftriaxone and gentamicin. Susceptibility testing showed most Gram-negative bacteria were ESBL producers and multi drug resistant (MDR).

### Antimicrobial Resistance Pattern for Bacteria Isolated From Blood Cultures

Antimicrobial resistance pattern among Gram-negative bacteria (GNB) and Gram-positive bacteria (GPB) is shown in Table 4. GNBdisplayed high rates of resistance to commonly used antimicrobials. Extended-spectrum beta-lactamase (ESBL) phenotype was observed in 51% of the GNB. *K. pneumoniae* displayed the highest rate of ESBL (90%) followed by *E. coli* (36%) and other Gram-negative bacteria (14%). Multidrug resistance (MDR), defined as resistance to three or more classes of antimicrobials, was also high (54%) among GNB. MDR among *K. pneumoniae*, *E. coli*, and *Acinetobacter* spp. were 90.3, 36, and 40%, respectively. Gentamicin resistance among GNB was found in 49%. One third of GNB were resistant to ciprofloxacin, and the highest resistance rate(44%) was among *K. pneumoniae.* Resistance to meropenem was observed in the following isolates of *A. baumannii* (*n* = 2) and two isolates of *Pseudomonas* spp. (*n* = 2). Like GNB, GPB also displayed high rates of resistance toward different antimicrobial agents. For *S. aureus*, the prevalence of Methicillin resistant *S. aureus* (MRSA) was 26%, and MDR was seen in one third of isolates. *Enterococcus* spp. showed a high rate of MDR of over 80%. There was no vancomycin resistance detected among *S. aureus* and *Enterococcus* spp.

**TABLE 4:** Antimicrobial resistance pattern for Gram-negative and Gram-positive bacteria isolated from children admitted with fever from March 2017 to July 2018 in Dar es Salaam, Tanzania.

**Antimicrobial agent**	**Gram-negative bacteria**						**Gram-positive bacteria**	
	***K. pneumoniae***	***E. coli***	***Acinetobacter* spp.**	***Pseudomonas* spp.**	Others*	**Total**	***S. aureus***	***Enterococcus* spp.**
	**n = 62**	**n = 28**	**n = 10**	**n = 12**	**n = 22**	**n = 134**	**n = 42**	**n = 30**
	**n (%)**	**n (%)**	**n (%)**	**n (%)**	**n (%)**	**n (%)**	**n (%)**	**n (%)**
PN	NT	NT	NT	NT	NT	NT	40 (95.2%)	17 (56.7%)
AMC	NT	NT	NT	NT	NT	NT	0	17 (56.7%)
AMP	NT	NT	NT	NT	NT	NT	NT	18 (60.0%)
CIP	27 (43.5)	11 (39.3)	2 (20.0)	2 (16.7)	2 (9)	44 (32.83)	10 (23.8%)	21 (70.0%)
TZP	40 (64.5)	0 (0.0)	4 (40.0)	0	0	44 (32.83)	NT	NT
TGC	22 (35.5)	1 (3.6)	NT	NT	3 (13.6)	26 (21.31)	NT	0
FOX	1 (1.6)	2 (7.1)	NT	NT	3 (13.6)	5 (4.09)	11 (26.2%)	NT
ATM	50 (80.6)	10 (35.7)	NT	1 (8.3)	2 (9)	63 (50.81)	NT	NT
CAZ	56 (90.3)	10 (35.7)	5 (50.0)	0	2 (9)	73 (54.5)	NT	NT
CTX	56 (90.3)	10 (35.7)	10 (100)	NT	2 (9)	78 (58.2)	11 (26.2%)	NT
SXT	57 (91.9)	22 (78.6)	4 (40.0)	NT	7 (31.8)	90 (73.77)	1 (2.4%)	NT
CN	53 (85.5)	7 (25.6)	4 (40.0)	1 (8.3)	1 (84.5)	66 (49.25)	14 (33.3%)	NT
C	14 (22.6)	4 (14.3)	NT	NT	2 (9)	20 (17.86)	4 (9.5%)	6 (20.0%)
DO	25 (40.3)	0 (0.0)	0	NT	4 (18)	29 (7.38)	10 (23.8%)	10 (33.3%)
LIN	NT	NT	NT	NT	NT	NT	NT	5 (16.7%)
MEM	0 (0.0)	0 (0.0)	2 (20.0)	2 (16.7)	0	4 (2.98)	7 (16.7%)	NT
VAN	NT	NT	NT	NT	NT	NT	0 (0.0)	0 (0.0)

Others* = Salmonella typhi and Salmonella spp., E. cloacae and Serratia spp.; PN, penicillin; AMC, amoxicillin/clavulanic acid; AMP, ampicillin; CIP, ciprofloxacin; TZP, piperacillin/tazobactam; TGC, tigecycline; FOX, cefoxitin; ATM, aztreonam; CAZ, ceftazidime; CTX, cefotaxime; SXT, sulfamethoxazole/trimethoprim; CN, gentamicin; C, chloramphenicol; DO, doxycycline; MEM, meropenem; LIN, linezolid; VAN, vancomycin; NT, not tested.

## Discussion

In an African setting, identifying etiologies of fever in severe BSI is challenging, first, because of lack of affordable diagnostic tools for conditions other than malaria, and second, fever is a non-specific symptom due to a panoply of conditions. This study demonstrated etiologies of severe febrile illness in 20% of children hospitalized in Dar es Salaam, Tanzania.

The frequent use of antibiotics before specimen for blood culture is taken can likely underestimate the incidence of BSI, especially for fastidious bacteria such as pneumococci, which are susceptible to commonly used antibiotics. In the present study, over three quarters of the children received antibiotics prior to blood culture. This is important to take into account when estimating the relative importance of different infectious agents in severe infections in children. Nevertheless, the prevalence of bacteraemia and fungemia obtained in this study is comparable to what was reported in children in Dar es Salaam (14%) 17 years ago (Blomberg et al., 2007) and in other low- and middle-income countries (11%) (Prasad et al., 2015). As previously reported (Blomberg et al., 2007), *K. pneumoniae* and *E. coli* were the most common bacteria isolated.

Dar es Salaam is the region of low to moderate malaria endemicity (Wang et al., 2006; D’Acremont et al., 2011). Despite using a very sensitive molecular method (Hofmann et al., 2015), our study found a low prevalence of malaria (10%) compared to 22% in the year 2001/2002 and 25% in 2009 using microscopy and PCR, respectively, in previous studies among children admitted with febrile illness in the Dar es Salaam region (Blomberg et al., 2005; Strom et al., 2013). The observed low malaria prevalence in this study provide further evidence of the potential benefits of national malaria control strategies in reducing malaria cases in Tanzania and elsewhere in sub-Saharan Africa (Mtove et al., 2011; Bhatt et al., 2015). Although some studies (Mackenzie et al., 2010; Mtove et al., 2011; Scott et al., 2011) have found that reduction of malaria cases occurs in parallel with reduction of invasive bacteria in BSI in children, our findings of the same prevalence of bacteraemia in Dar es Salaam as many years back, does not support such parallel decline. This may partly be explained by the finding that malaria has been associated with bacteraemia caused by non-typhoid Salmonella (Takem et al., 2014), which is not common in the present study setting.

We found significant association between the quantity of *P. falciparum* genomes in blood and the most severe form of clinical malaria with three or more severity criteria. This concurs with the findings of a multicenter study by Imwong et al. (2015). The findings of this study and that of Imwong et al. (2015) suggest that the use of plasma qPCR could help to differentiate severe from non-severe malaria, and if possible, guide management of severe BSI caused by malaria. In addition, 40% of children with malaria-bacteria co-infection had severe malaria, as well as high parasite load of *P. falciparum* in plasma. These findings agree with that of Berkley et al. (2009) and Nadjm et al. (2010) who have previously reported association between high malaria parasitaemia and bacterial co-infection.

We report a high rate of AMR among Gram-negative bacteria, which is in agreement with previous findings (Blomberg et al., 2007). In addition, we found a significant increase of ESBL producing Gram-negative bacteria (50.7%) compared to a previous study (15%, *P* < 0.001) in the same study setting (Blomberg et al., 2005). Most bacteria were MDR, leaving limited treatment options available. The frequent use of antibiotics including third generation cephalosporins following empirical treatment guideline might have promoted an increase in antimicrobial resistance. It is worrisome that among *K. pneumoniae*, which is the commonest cause of BSI, 90% are ESBL producers as well as being resistant to gentamicin. This means that penicillins, cephalosporins, and aminoglycosides, which are the most frequently used drugs for the treatment of BSI, are inappropriate. The finding of high in-hospital death of *Klebsiella* bloodstream infection in our study supports previous findings (Zaidi et al., 2005; Blomberg et al., 2007).

The majority of children (89%) who received antibiotics on admission did not have growth in blood culture. A high proportion of children were given an antibiotic on admission because the study setting is a low-income country, and availability of laboratory tests such as blood culture to confirm for the bacterial etiology of fever is limited to a few referral hospitals. As a result, initial use of antibiotics following empirical treatment guidelines by the clinicians is difficult to avoid, in an attempt to save lives of children with life threatening severe BSI. This massive use of antibiotics will contribute to increase the existing problem of antimicrobial resistance in this study setting.

In the present study the in-hospital mortality was 4%. This is much lower than was reported 17 years ago by Blomberg et al. (2007) (34.9%) in the same city. One reason for such as big difference could be that the previous study included only children admitted to the tertiary hospital (MNH), indicating that patients were severely ill and likely were referred late from other hospitals. In the present study the majority of children were included from regional hospitals, and a minority from the referral national hospital. Even in the present study, children admitted at the tertiary level hospital had a higher risk of death than children admitted to lower level hospitals. Other reasons for low in-hospital death could be a general reduction in under-five mortality attributed to expanded malaria control programs including availability of effective malaria treatment drugs such as ACTs in Tanzania (Smithson et al., 2015). Furthermore, the pneumococcal vaccination program and a reduction in HIV transmission may also have contributed to the reduction in under-five mortality. In-hospital mortality was significantly higher among bacteraemic children compared to children with malaria. This is in agreement with previous findings in Tanzania and Kenya and implies that survival of children with BSI requires prompt antimicrobial treatment (Berkley et al., 2005; Blomberg et al., 2007). In the present study, only four children with malaria died and three of these children received appropriate anti-malarial treatment. These children may have died because they presented too late at the hospital, they were unconscious, which was found as an independent risk factor for death in the study, and as a result they could not benefit from the malaria treatment they received. Bacteraemia was an independent risk factor for in-hospital mortality, as previously reported (Blomberg et al., 2005; Bassat et al., 2009; Nadjm et al., 2010). Furthermore, over two thirds of bacteraemic children who died were infected with Gram-negative bacteria, concurrent with other studies (Blomberg et al., 2007). Sixteen out of 18 (88%) of the children died with bacteraemia despite receiving antibiotics. This could be because most of the bacteria isolated were resistant to given regimens, and treatment they received was thus inappropriate.

This study has some limitations. First, our study was able to identify etiologies of severe febrile illness for only 20% of participants, because viruses and zoonotic bacteria were not investigated for in this study. Viruses were found to be the most common cause of fever in children attending outpatient clinics in Dar es Salaam (D’Acremont et al., 2011). Whether this is also the case in admitted children requires further analysis. In a study from the northern part of Tanzania, many children admitted with fever had zoonotic infections such as leptospirosis, brucellosis, Q-fever, and rickettsiosis (Crump et al., 2013). Second, diagnosis of BSI due to specific syndromes such as pneumonia, meningitis, UTI, gastroenteritis, and upper respiratory tract infections, which would require analysis of additional specimens such as CSF, urine, sputum culture, chest x-ray, and nasopharyngeal swabs, was not performed in this study. Third, we found that HIV infection increased risk of in-hospital death in the univariable analysis. However, the missing data on HIV results made it impossible for HIV variable to be included in multivariable analysis, and hence we could not confirm or rule out HIV infection as a cause of in-hospital death. HIV-DNA PCR was not performed to confirm the rapid HIV antibody test for infants below 18 months. Last, we did not have outcomes of 80 children whose results may have changed the fatality data presented.

In summary, compared to previous studies in this region, our study showed a reduction in malaria prevalence, a decrease in in-hospital mortality, and an increase in antimicrobial resistance (AMR) including ESBLs and multidrug resistance. A decrease in mortality may be attributed to the on-going implemented malaria control programs in the country, and the implementation of artemisinin-based treatment regimens may also have contributed. A reduction of congenital HIV may also have contributed to the reduced mortality. An increase of AMR highlights the importance of continued strengthening of diagnostic capability and antimicrobial stewardship programs. We also found malaria and bacteraemia contributed equally in causing febrile illness, but bacteraemia caused higher in-hospital death. The most severe form of clinical malaria was associated with *P. falciparum* parasitaemia.

## Data Availability Statement

The raw data supporting the conclusions of this article will be made available by the authors, without undue reservation, to any qualified researcher.

## Ethics Statement

The studies involving human participants were reviewed and approved by the Senate Research and Publications Committee of Muhimbili University of Health and Allied Sciences, National Institute of Medical Research in Tanzania and from the Regional Committee for Medical and Health Research Ethics (REK) in Western Norway. Written informed consent to participate in this study was provided by the participants’ legal guardian/next of kin.

## Author Contributions

SJM, NL, and BB conceived the study. SJM, NL, BB, JM, NM, SA, and KMa contributed to designing the study. SJM, JM, and NM involved in data collection. SJM and JM performed the microbiological investigations. SJM and KH set up qPCR and analysis for *P. falciparum*. SJM performed statistical analysis and drafted the manuscript. All authors contributed to writing the manuscript and approved the final version.

## Conflict of Interest

The authors declare that the research was conducted in the absence of any commercial or financial relationships that could be construed as a potential conflict of interest.
