# Supplementary material for: Bacteraemia, Malaria, and Case Fatality Among Children Hospitalized With Fever in Dar es Salaam, Tanzania
Source: Front Microbiol. 2020 Sep 10;11:2118. doi: 10.3389/fmicb.2020.02118 (PMC7511546; doi:10.3389/fmicb.2020.02118)
Supplement: Supplementary file 1 [file Table_1.docx]

**Supplementary Table 1: Univariable analyses of bacteraemia/fungaemia and malaria and social demographic/clinical characteristics of children admitted with fever from March 2017 to July 2018 in Dar- es Salaam, Tanzania**

| **Demographic/**  **Clinical Characteristics** | **N** | **n (%)** | **Malaria (n=2063)** | **N** | **n (%)** | **Bacteraemia/fungaemia (n=2226)** |
| --- | --- | --- | --- | --- | --- | --- |
|  |  |  | **P-value; OR (95% CI)** |  |  | **P-value; OR (95% CI)** |
| **Age in months** |  |  |  |  |  |  |
| 0-3 | 1115 | 31 (2.8) | 1 | 1214 | 172 (14.2) | **<0.001; 2.58 (1.63-4.09)** |
| 4-6 | 111 | 10 (9.0) | **0.001; 3.46 (1.65-7.27)** | 122 | 10 (8.2) | 0.4; 1.39 (0.64-3.04) |
| 7-11 | 212 | 15 (7.1) | **0.003; 2.66 (1.41-5.02)** | 220 | 12 (5.5) | 0.78; 0.9 (0.44-1.86) |
| 12-24 | 285 | 39 (13.7) | **<0.001; 5.54 (3.39-9.1)** | 304 | 20 (6.6) | 0.76; 1.1 (0.59-2.06) |
| ≥ 25 | 376 | 116 (30.9) | **<0.001; 16.5 (10.8-25.2)** | 366 | 22 (6.0) | 1 |
| **Sex** |  |  |  |  |  |  |
| Male | 1205 | 127 (10.5) | 0.24;1.19 (0.89-1.61) | 1299 | 143 (11.0) | 0.46;0.9 (0.68-1.19) |
| Female | 858 | 77 (9.0) | 1 | 927 | 93 (10.0) | 1 |
| **HIV status^*^** |  |  |  |  |  |  |
| Positive | 37 | 6 (16.2) | 0.97;0.98 (0.4-2.39) | 40 | 1 (2.5) | 0.29;0.34 (0.05-2.52) |
| Negative | 1040 | 171 (16.4) | 1 | 1116 | 78 (7.0) | 1 |
| **Consciousness** |  |  |  |  |  |  |
| Unconscious | 119 | 32 (26.9) | **<0.001;3.79 (2.45-5.85)** | 136 | 18 (13.2) | 0.31;1.31 (0.78-2.19) |
| Awake | 1944 | 172 (8.8) | 1 | 2090 | 218 (10.4) | 1 |
| **Anaemia^*^ g/dL** |  |  |  |  |  |  |
| Hb below 5 | 26 | 10 (38.5) | **<0.001;8.14 (3.56-18.61)** | 27 | 2 (7.4) | 0.91;0.92 (0.21-3.94) |
| HB above 5 | 982 | 70 (7.1) | 1 | 1010 | 81 (8.0) | 1 |
| **Diarrhoea** |  |  |  |  |  |  |
| Yes | 225 | 22 (9.8) | 0.95;0.99 (0.62-1.57) | 243 | 23 (9.5) | 0.54;0.87 (0.55-1.37) |
| No | 1838 | 182 (9.9) | 1 | 1983 | 213 (10.7) | 1 |
| **Cough** |  |  |  |  |  |  |
| Yes | 417 | 34 (8.2) | 0.18;0.77 (0.52-1.13) | 445 | 35 (7.9) | 0.04;0.67 (0.46-0.98) |
| No | 1646 | 170 (10.3) | 1 | 1781 | 201 (11.3) | 1 |
| **Convulsions** |  |  |  |  |  |  |
| Yes | 307 | 54 (17.6) | **<0.001;2.28 (1.63-3.21)** | 339 | 35 (10.3) | 0.86;0.97 (0.66-1.41) |
| No | 1756 | 150 (8.5) | 1 | 1887 | 201 (10.7) | 1 |
| **Vomiting** |  |  |  |  |  |  |
| Yes | 303 | 55 (18.2) | **<0.001;2.39 (1.71-3.36)** | 331 | 30 (9.1) | 0.31;0.82 (0.55-1.22) |
| No | 1760 | 149 (8.5) | 1 | 1895 | 206 (10.9) | 1 |
| **Jaundice** |  |  |  |  |  |  |
| Yes | 59 | 10 (16.9) | 0.07;1.9 (0.95-3.82) | 63 | 8 (12.7) | 0.58;1.23 (0.58-2.62) |
| No | 2004 | 194 (9.7) | 1 | 2163 | 228 (10.5) | 1 |
| **Neck stiffness** |  |  |  |  |  |  |
| Yes | 53 | 10 (18.9) | **0.03;2.18 (1.08-4.40)** | 59 | 4 (6.8) | 0.34;0.61 (0.22-1.69) |
| No | 2010 | 194 (9.7) | 1 | 2167 | 232 (10.7) | 1 |

Note: OR=odds ratio; CI=confidence interval; Confidence intervals that do not overlap the null value of OR=1 are shown in bold; * Anaemia n=1008, *HIV-status n=1077
